# Serum cytokine profiles at near term-equivalent age and their association with neurodevelopmental outcomes in preterm infants: an exploratory study

**DOI:** 10.3389/fped.2025.1667521

**Published:** 2025-08-28

**Authors:** Yan-Jun Wu, Wei-Chi Wu, Shih-Ming Chu, Reyin Lien, Ming-Chou Chiang, Chien-Chung Lee

**Affiliations:** ^1^ Department of Pediatrics, Division of Neonatology, Chang Gung Memorial Hospital, School of Medicine, Chang Gung University, Taoyuan, Taiwan; ^2^ Department of Pediatrics, Taoyuan Armed Forces General Hospital, Taoyuan, Taiwan; ^3^ Department of Ophthalmology, Chang Gung Memorial Hospital, School of Medicine, Chang Gung University, Taoyuan, Taiwan

**Keywords:** biomarkers, preterm, neurodevelopment, IFN-γ, IL-17

## Abstract

**Background:**

While early-life cytokine profiles have been linked to neurodevelopmental outcomes in preterm infants, their prognostic value is limited by clinical instability and inflammatory comorbidities in the immediate postnatal period. This study explores cytokine levels measured during a more stable developmental window—near term-equivalent age [postmenstrual age (PMA) 34–38 weeks]—and their association with neurodevelopmental outcomes.

**Methods:**

We prospectively enrolled 35 preterm infants (birth weight, 500–1,500 g). Serum cytokine levels were measured at PMA 34, 36, and 38 weeks. Neurodevelopment was assessed at 12 months’ corrected age using standardized tools (BSID-III). Infants were classified into neurodevelopmental impairment (NDI) and non-NDI groups. Cytokine levels and their changes were compared between groups.

**Results:**

Elevated IFN-γ levels at PMA 34 weeks were associated with a higher risk of NDI. Conversely, higher levels of Eotaxin-2, IL-2, IL-11, IL-16, MIP-1δ, PDGF-BB, TIMP-2, and TNF-β at PMA 36–38 weeks were observed more frequently in the non-NDI group. The trends also differed: increased IL-17 and decreased Eotaxin-1, Eotaxin-2, IL-7, IL-16, MIP-1α, MIP-1β, PDGF-BB, and TIMP-2 between PMA 34–36 weeks, and further declines in ICAM-1, IL-7, MIP-1α, and MIP-1β by PMA 38 weeks were associated with adverse outcomes. All identified biomarkers demonstrated good discriminatory ability, particularly changes in Eotaxin-2 between PMA 34 and 36 weeks and PDGF-BB between PMA 34 and 38 weeks.

**Conclusions:**

Serum cytokine levels and their trajectories during PMA 34–38 weeks may serve as potential biomarkers for identifying preterm infants at risk of neurodevelopmental impairment. Further studies with larger cohorts are needed to clarify their interplay with preterm morbidities.

## Highlights

• Near-term cytokines help identify preterm infants at neurodevelopment risk.
• Most cytokines predict good neurodevelopment; only IFN-γ and IL-17 predict poor outcomes.
• Both cytokine levels and their changes may predict outcomes in preterm infants.
• Preterm comorbidities impact biomarker levels at near-term age.

## Introduction

Premature infants are at an increased risk of developing varying degrees of neurodevelopmental impairment (NDI) (1, 2). In these infants, fetal growth restriction has been identified as an independent risk factor (3). Studies suggest correlations between specific blood biomarkers and neurodevelopmental outcomes in infants. For instance, elevated levels of proinflammatory cytokines, including interleukin (IL)-1β, IL-8, IL-9, tumor necrosis factor (TNF)-α, and regulated upon activation, normal T cell expressed and secreted (RANTES), along with decreased levels of anti-inflammatory cytokines, such as IL-2 and IL-3, within the first day after birth, have shown good sensitivity and specificity in predicting cerebral palsy (CP) in late preterm and term infants (4). Another cohort study conducted on 1,067 extremely low birth weight (≤1,000 g) infants found that IL-8 levels were elevated during the initial four days and remained higher in infants who subsequently developed CP (5).

However, some studies also have provided different insights, suggesting that cytokines in early postnatal age may not effectively predict neurologic outcomes. A study that collected cord blood samples at birth from 400 neonates found no association between elevated levels of cord serum IL-6, C-Reactive Protein (CRP), and Myeloperoxidase (MPO) at birth and poor neurodevelopmental outcomes (6). In a nested case-control study of 615 preterm infants with a gestational age (GA) between 24 and 31 6/7 weeks, cord serum IL-8, IL-1β, and TNF-α levels were not associated with subsequent CP or neurodevelopmental delay at the 2-year follow-up (7). Additionally, a study that measured cytokines on average 2.4 days postnatally in 271 very preterm infants born before 32 weeks GA found no associations between 11 cytokines [IL-1, −2, −4, −5, −6, −8, −10, and −12; granulocyte–macrophage colony-stimulating factor (GMCSF); interferon (IFN)-γ; and TNF-α] and later diagnosis of CP (8).

Conflicting research results may be attributed to the fact that preterm infants often face significant clinical instability during the initial weeks of life. This period may be affected by various pathological insults that can alter their physiological condition. As a result, cytokines in the early postnatal period may not provide strong neurological prognostic indicators. Limited studies have hinted that the relationship between various blood biomarkers and neurodevelopmental outcomes may vary depending on the specific postnatal time points and the types of specimens evaluated. A large cohort study of 1,506 premature infants born at a GA of less than 28 weeks found that serum biomarkers such as CRP, IL-8, and intercellular adhesion molecule-1 (ICAM-1), measured on postnatal days 21 and 28, were associated with mental or psychomotor developmental impairments in these infants (9). In another study of 51 term infants, higher serum levels of IL-1β at six months of age predicted decreased motor skill performance at 30 months of age (10).

Therefore, we investigated cytokine concentrations at postmenstrual ages (PMA) of 34, 36, and 38 weeks—a period considered to represent near term-equivalent age and characterized by relatively stable clinical conditions—to explore potential associations between these biomarkers and neurodevelopmental outcomes.

## Methods

### Study participants

This prospective cohort study was conducted in the neonatal intensive care units of our hospital between December 1, 2019, and June 30, 2022. Preterm infants with birth weights between 500 g and 1,500 g were enrolled after written informed consent was obtained from their parents. Exclusion criteria included major congenital anomalies, such as chromosomal abnormalities or central nervous system malformations. Infants were also excluded from the final analysis if they died before serum cytokine sampling, had incomplete medical records, or lacked neurodevelopmental assessment data. The study was approved by the Institutional Review Board of our hospital (approval number: 201902088A3).

### Data collection

Demographic data were retrospectively collected from electronic medical records, including maternal characteristics [e.g., mother's age, pregnancy complications such as gestational diabetes mellitus (GDM), premature rupture of membranes (PROM), pre-eclampsia/eclampsia], infant baseline information (e.g., GA, birth weight, APGAR scores), and clinical outcomes (e.g., severe intraventricular hemorrhage (IVH), hemodynamically significant patent ductus arteriosus (HsPDA), necrotizing enterocolitis (NEC), Grade II/III bronchopulmonary dysplasia (BPD). Severe IVH was defined as IVH grade ≥3 on intracranial ultrasound. HsPDA was defined as cases requiring medical or surgical ligation. BPD was diagnosed basing on the 2019 Jensen definition (11). Interventions, including postnatal use of intravenous or inhaled steroids and exclusive breast milk feeding, were included in the analysis. Nutritional status was assessed by examining changes in Z-scores from birth to discharge. For infants discharged at a PMA of less than 50 weeks, the Fenton Growth Chart (2013) was used (12), while the 2006 WHO Growth Standards were applied for those discharged at 50 weeks or later (13).

### Neurodevelopment assessment

The Bayley Scales of Infant and Toddler Development, Third Edition (BSID-III) were used to assess neurodevelopmental condition at 12 months of corrected age (14). The BSID-III includes domains for cognitive (91 items), language (97 items), motor (138 items), social-emotional (35 items), and adaptive behavior (241 items). Only the first three domains were included in the analysis. Neurodevelopmental impairment (NDI) was defined as a BSID-III score <70 in any of the motor, cognitive, or language domains at 12 months of corrected age. Infants were classified into NDI and non-NDI groups.

### Cytokines analysis

Blood samples were collected three times: at 34 weeks PMA, 36 weeks PMA, and 38 weeks PMA, with a 2-week interval between collections. After reviewing the cytokines reported to correlate with neurodevelopment in preterm infants, we selected a commercially available multiplex assay (Quantibody® Human Inflammation Array 3; RayBiotech, Peachtree Corners, GA, USA) to analyze serum cytokine levels at each time point. This assay quantifies 40 cytokines, including B-lymphocyte chemoattractant (BLC), eotaxin-1, eotaxin-2, granulocyte colony-stimulating factor (GCSF), GMCSF, I-309, ICAM-1, IFN-γ, IFN-1α, IFN-1β, IL-1Rα, IL-2, IL-4, IL-5, IL-6, IL-6R, IL-7, IL-8, IL-10, IL-11, IL-12p40, IL-12p70, IL-13, IL-15, IL-16, IL-17, monocyte chemoattractant protein (MCP)-1, macrophage colony-stimulating factor (MCSF), monokine induced by IFN-γ (MIG), macrophage inflammatory protein (MIP)-1α, MIP-1β, MIP-1δ, platelet-derived growth factor-BB (PDGF-BB), RANTES, tissue inhibitor of metalloproteinase (TIMP)-1, TIMP-2, TNF-α, TNF-β, TNF receptor (TNFR)-1, and TNFR-2, including several previously reported to be significant. Serum cytokine levels at each time point, as well as the cytokine changes between each interval, were compared between two groups.

### Statistical analysis

In comparing demographic variables, a Chi-square test was used to assess differences between categorical variables, and the independent Student's *t*-test was applied to analyze continuous, normally distributed variables, which are presented as means and standard deviations (SDs). Serum cytokine levels at each time point and cytokine changes between intervals were compared using the nonparametric Mann–Whitney *U*-test between two groups. The area under the ROC curve (AUC) was calculated for significant biomarkers. The generalized estimating equations approach was used to identify factors associated with changes in serum cytokine levels. A significant difference was considered for *p*-values less than 0.05. Statistical analyses were performed using IBM SPSS Statistics (version 27.0, Armonk, NY: IBM Corp).

## Results

**Figure 1:**
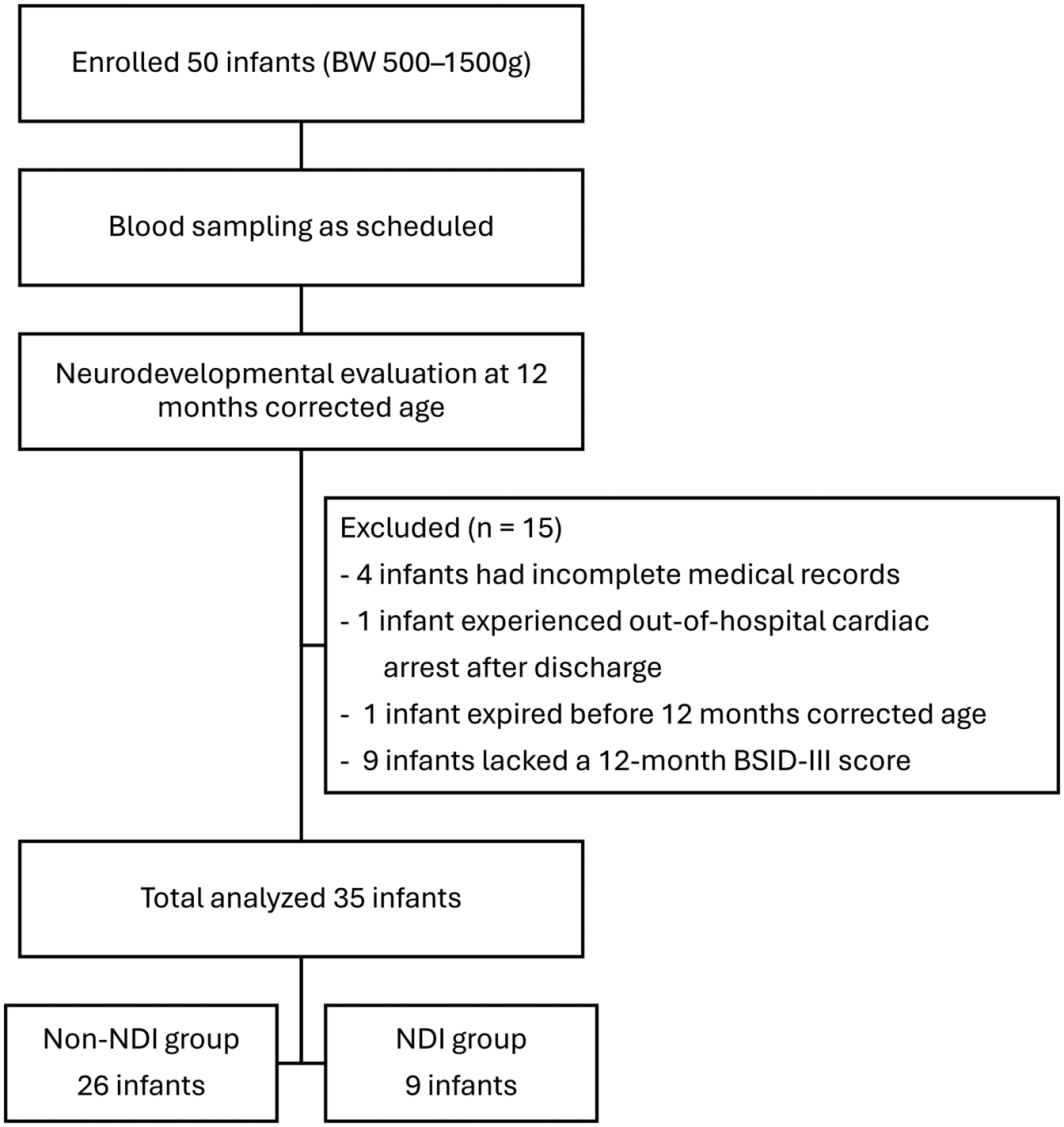
Flowchart of the study design, participant selection, and final inclusion.

A total of 50 premature infants were enrolled in this study. Fifteen infants were excluded for the following reasons: four had incomplete medical records, one expired before the neurodevelopmental assessment, one experienced an out-of-hospital cardiac arrest after discharge, and nine lacked a 12-month BSID-III score. Thirty-five preterm infants were included in the final analysis, with 26 in the non-NDI group and 9 in the NDI group (Figure 1). The characteristics of these two groups were presented in Table 1. There were no significant differences in maternal and infant characteristics between the two groups.

**Table 1:** Maternal and infant characteristics between infants with and without neurodevelopmental impairment.

Demographic data	Non-NDI (*N* = 26)	NDI (*N* = 9)	*P*
Maternal characteristics			
Mother's age, years (mean ± SD)	33 ± 5	35 ± 5	0.459
Pre-eclampsia or eclampsia, *n* (%)	3 (11.5)	2 (22.2)	0.586
Prom >18 hr, *n* (%)	1 (3.8)	0	1.000
Gdm, *n* (%)	0	1 (11.1)	0.257
Antenatal MgSO4, *n* (%)	22 (84.6)	8 (88.9)	1.000
Antenatal steroids, *n* (%)	25 (96.2)	9 (100)	1.000
Perinatal antibiotics, *n* (%)	18 (69.2)	7 (77.8)	1.000
Infant characteristics			
Gestational age, weeks (mean ± SD)	27 ± 2	27 ± 3	0.920
Birth weight, g (mean ± SD)	933 ± 278	912 ± 285	0.849
Male, *n* (%)	14 (53.8)	6 (66.7)	0.700
Multiple birth, *n* (%)	8 (80.8)	1 (11.1)	0.391
Cesarean section, *n* (%)	19 (73.1)	7 (77.8)	1.000
APGAR score 1 min, (mean ± SD)	6 ± 1	4 ± 3	0.051
APGAR score 5 minutes, (mean ± SD)	8 ± 1	7 ± 2	0.076
Early-onset sepsis, *n* (%)	1 (3.8)	0	1.000
Late-onset sepsis, *n* (%)	3 (11.5)	4 (44.4)	0.055
IVH ≧ grade 3, *n* (%)	2 (7.7)	1 (11.1)	1.000
Necrotizing enterocolitis^a^, *n* (%)	1 (3.8)	0	1.000
HsPDA, *n* (%)	18 (69.2)	3 (33.3)	0.112
Bronchopulmonary dysplasia^b^, *n* (%)	23 (88.5)	9 (100)	1.000
Use of intravenous steroids, *n* (%)	2 (7.7)	0	1.000
Use of inhalation steroids, *n* (%)	11 (42.3)	3 (33.3)	0.712
Exclusive breast milk use, *n* (%)	7 (26.9)	2 (22.2)	1.000
△Z-score change from birth to discharge, (mean ± SD)	−1.00 ± 0.88	−1.63 ± 1.28	0.11

Values are presented as mean ± standard deviation or number (%).

GDM, gestational diabetes mellitus; HsPDA, hemodynamic significant patent ductus arteriosus, defined as requiring medical or surgical ligation; IVH, intraventricular hemorrhage; NDI: neurodevelopment impairment; PROM, premature rupture of membrane.

^a^ Bell stage ≥IIa.

^b^ 2019 Jensen definition ≥grade II.

When analyzing the serum cytokine levels at each time point, we identified 9 cytokines with significant differences between the two groups, potentially linked to neurodevelopmental outcomes, as shown in Table 2. Higher serum levels of IFN-γ were observed in the NDI group at 34 weeks PMA, suggesting a potentially detrimental effect on neurodevelopment. Conversely, the non-NDI group exhibited higher serum levels of eight other cytokines—Eotaxin-2, IL-2, IL-11, IL-16, MIP-1δ, PDGF-BB, TIMP-2, TNF-β—at 36 weeks PMA or 38 weeks PMA, indicating a potentially favorable effect. The levels of all 40 cytokines at each time point were compared and summarized in Supplementary Table 1. No significant differences were observed for the other 31 cytokines at any time point between the two groups.

**Table 2:** Cytokine levels with significant differences at each time point between infants with and without neurodevelopmental impairment.

Cytokines (pg/ml)	PMA 34 weeks		*P*	PMA 36 weeks		*P*	PMA 38 weeks		*P*
	Non-NDI	NDI		Non-NDI	NDI		Non-NDI	NDI	
Detrimental									
IFN-γ	1.59 (0.00, 6.48)	4.44 (0.42, 9.04)	**0**.**025**	1.28 (0.00, 78.62)	2.15 (0.00, 98.75)	0.255	2.06 (0.00, 331.09)	2.17 (0.00, 166.73)	0.565
Favorable									
Eotaxin-2	301.47 (33.10, 515.85)	325.99 (46.13, 690.73)	0.697	336.28 (52.43, 624.08)	130.49 (58.34, 388.64)	**0**.**023**	322.28 (81.82, 669.78)	191.54 (136.15, 437.53)	0.224
IL-2	19.11 (0.12, 72.41)	13.82 (0.00, 54.36)	0.516	19.90 (4.03, 46.06)	8.28 (0.00, 29.98)	0.086	21.49 (5.19, 140.18)	14.04 (1.44, 22.42)	**0**.**042**
IL-11	96.67 (0.00, 374.27)	75.01 (0.00, 192.15)	0.670	62.04 (0.00, 756.31)	59.56 (0.00, 164.19)	0.725	86.40 (0.00, 543.21)	17.82 (0.00, 187.47)	**0**.**028**
IL-16	189.92 (5.98, 1,099.50)	509.29 (45.53, 1,142.25)	0.239	389.70 (13.82, 1,023.00)	50.82 (8.40, 409.72)	**0**.**034**	350.62 (12.17, 1,179.41)	56.13 (11.32, 965.40)	0.079
MIP-1δ	301.27 (187.43, 496.58)	256.74 (169.40, 302.41)	0.079	282.19 (154.84, 455.66)	262.04 (112.50, 344.14)	0.073	316.77 (143.57, 474.35)	246.99 (147.89, 295.22)	**0**.**018**
PDGF-BB	17,393.53 (1,472.78, 22,385.23)	17,210.16 (4,235.79, 24,451.61)	0.643	17,368.56 (6,700.26, 23,205.80)	11,014.52 (2,059.40, 20,522.66)	**0**.**025**	17,843.52 (8,301.64, 24,273.24)	15,539.39 (3,049.97, 22,588.55)	0.119
TIMP-2	5,079.46 (2,130.64, 6,519.09)	4,732.85 (3,828.42, 6,505.92)	0.670	5,456.47 (3,771.14, 6,688.59)	4,124.53 (3,593.22, 5,773.04)	0.051	5,558.79 (3,491.90, 6,786.18)	4,675.14 (3,766.40, 5,882.53)	**0**.**042**
TNF-β	452.87 (13.22, 1,385.33)	451.39 (0.00, 1,026.14)	0.616	398.10 (0.00, 1,087.44)	142.41 (0.00, 550.87)	**0**.**031**	402.58 (57.76, 2,003.58)	303.76 (74.85, 1,162.25)	0.382

Bold values in the table indicate statistical significance.

Subsequently, we investigated the changes in serum cytokine levels between each time point and identified 10 cytokine changes associated with neurodevelopmental outcomes, which were summarized in Table 3. Elevated IL-17 levels, as well as decreased Eotaxin-1, Eotaxin-2, IL-7, IL-16, MIP-1α, MIP-1β, PDGF-BB, and TIMP-2 levels between PMA 34 and PMA 36 weeks, correlated with poor neurodevelopmental outcomes. Besides, decreased in ICAM-1, IL-7, IL-16, MIP-1α, and MIP-1β levels between PMA 34 and PMA 38 weeks was associated with poor neurodevelopmental outcomes. The comparisons of cytokine level changes across all 40 cytokines in each interval were listed in Supplementary Table 2.

**Table 3:** Significant changes in cytokine levels at each time interval between infants with and without neurodevelopmental impairment.

Cytokines (pg/ml)	PMA 34 weeks to PMA 36 weeks		*P*	PMA 36 weeks to PMA 38 weeks		*P*	PMA 34 weeks to PMA 38 weeks		*P*
	Non-NDI	NDI		Non-NDI	NDI		Non-NDI	NDI	
Detrimental									
IL-17	−0.54 (−49.62, 44.58)	4.30 (−6.39, 21.55)	**0**.**031**	0.73 (−44.26, 73.60)	−0.74 (−11.12, 23.66)	0.255	0.54 (−49.73, 67.63)	0.00 (−2.86, 33.83)	0.956
Favorable									
Eotaxin-1	38.65 (−213.34, 334.51)	−119.21 (−409.37, 74.67)	**0**.**009**	−14.28 (−254.04, 341.17)	30.37 (−184.13, 214.3)	0.362	11.97 (−468.38, 371.14)	−60.46 (−589.30, 48.16)	0.056
Eotaxin-2	55.26 (−221.93, 291.60)	−101.61 (−437.87, 19.79)	**0**.**002**	1.00 (−172.19, 340.09)	26.74 (−120.04, 347.29)	0.305	19.58 (−181.32, 578.05)	−90.58 (−477.94, 95.24)	0.079
ICAM-1	−17.02 (−962.25, 1,357.59)	−252.85 (−1,236.46, 300.49)	0.119	−67.34 (−1,058.45, 1,718.56)	−137.64 (−1,005.44, 427.65)	0.781	−69.09 (−1,069.27, 756.30)	−399.92 (−808.81, 151.82)	**0**.**010**
IL-7	20.59 (−147.68, 301.48)	−23.40 (−126.01, 2.40)	**0**.**020**	15.97 (−108.42, 158.79)	5.08 (−54.80, 240.85)	0.956	19.71 (−88.17, 243.35)	−23.01 (−93.00, 171.33)	**0**.**046**
IL-16	13.12 (−746.42, 991.24)	−114.10 (−1,098.75, 124.71)	**0**.**038**	−4.00 (−604.31, 1,133.38)	5.31 (−78.58, 555.68)	0.565	60.18 (−1,024.98, 1,106.11)	−181.60 (−1,076.70, 680.39)	0.056
MIP-1α	2.64 (−143.82, 267.24)	−36.32 (−329.90, 13.44)	**0**.**038**	7.53 (−83.80, 979.56)	4.65 (−38.12, 212.40)	0.697	10.11 (−90.15, 835.74)	−50.30 (−292.52, 225.84)	**0**.**031**
MIP-1β	−4.13 (−55.47, 21.14)	−17.60 (−101.79, 1.06)	**0**.**007**	1.05 (−17.78, 194.45)	3.06 (−15.56, 49.53)	0.810	3.37 (−47.12, 172.56)	−16.68 (−82.45, 7.26)	**0**.**011**
PDGF-BB	1,684.09 (−10,464.61, 13,902.01)	−351,149 (−11,691.39, 754.14)	**0**.**004**	702.33 (−6,360.10, 10,301.67)	1,568.61 (−3,308.42, 14,759.51)	0.446	1,306.09 (−8,018.05, 12,897.32)	−3,028.39 (−10,927.76, 14,054.45)	0.056
TIMP-2	90.80 (−743.86, 3,959.80)	−383.73 (−1,050.79, 1,040.19)	**0**.**042**	232.24 (−2,332.42, 2,859.74)	173.18 (−174.05, 737.77)	0.926	413.94 (−1,332.68, 4,330.47)	−126.46 (−1,196.50, 898.53)	0.239

Bold values in the table indicate statistical significance.

The AUC for each significant biomarker was summarized in Table 4. Biomarkers with a detrimental effect indicating NDI showed an AUC of 0.752 for IFN-γ at PMA 34 weeks and an AUC of 0.744 for the change in IL-17 from PMA 34 weeks to 36 weeks. Biomarkers with a favorable effect also demonstrated good discriminatory ability, with all AUC values above 0.7. Notably, the change in Eotaxin-2 between PMA 34 and 36 weeks had an AUC of 0.833, and PDGF-BB between PMA 34 and 38 weeks had an AUC of 0.821.

**Table 4:** Area under the ROC curve (AUC) for detrimental cytokines predicting poor neurodevelopmental outcomes and favorable cytokines predicting absence of neurodevelopmental impairment.

Cytokines (pg/ml)	Time or interval	AUC	*P* value
Neurodevelopmental impairment			
IFN-γ	PMA 34 weeks	0.752	0.026
IL-17	PMA 34 weeks to PMA 36 weeks	0.744	0.031
Absence of neurodevelopmental impairment			
Eotaxin-2	PMA 36 weeks	0.756	0.024
IL-16	PMA 36 weeks	0.739	0.035
PDGF-BB	PMA 36 weeks	0.752	0.026
TNF-β	PMA 36 weeks	0.744	0.031
IL-2	PMA 38 weeks	0.731	0.042
IL-11	PMA 38 weeks	0.746	0.030
MIP-1δ	PMA 38 weeks	0.765	0.019
TIMP-2	PMA 38 weeks	0.731	0.042
Eotaxin-1	PMA 34 weeks to PMA 36 weeks	0.791	0.010
Eotaxin-2	PMA 34 weeks to PMA 36 weeks	0.833	0.003
IL-7	PMA 34 weeks to PMA 36 weeks	0.761	0.021
IL-16	PMA 34 weeks to PMA 36 weeks	0.735	0.038
MIP-1α	PMA 34 weeks to PMA 36 weeks	0.735	0.038
MIP-1β	PMA 34 weeks to PMA 36 weeks	0.799	0.008
PDGF-BB	PMA 34 weeks to PMA 36 weeks	0.821	0.005
TIMP-2	PMA 34 weeks to PMA 36 weeks	0.731	0.042
ICAM-1	PMA 34 weeks to PMA 38 weeks	0.786	0.011
IL-7	PMA 34 weeks to PMA 38 weeks	0.726	0.045
MIP-1α	PMA 34 weeks to PMA 38 weeks	0.744	0.031
MIP-1β	PMA 34 weeks to PMA 38 weeks	0.782	0.013

To assess the possible factors contributing to cytokine changes during this period, a generalized estimating equations approach was applied, and the results were summarized in Table 5. From PMA 34 weeks to 36 weeks, infants with severe IVH showed increased levels of IL-2, IL-7, and IL-11, while HsPDA was associated with increased levels of IL-11 and PDGF-BB. On the other hand, NEC was associated with decreased levels of Eotaxin-1, Eotaxin-2, IL-2, IL-16, MIP-1β, and PDGF-BB, and GA was associated with decreased levels of ICAM-1. From PMA 36 weeks to 38 weeks, only HsPDA was associated with increased levels of IL-11, while infants with LOS showed decreased levels of Eotaxin-1, IL-2, and IL-11. Additionally, NEC was associated with decreased levels of Eotaxin-1, Eotaxin-2, IL-2, IL-16, PDGF-BB, and TIMP-2, BPD with decreased MIP-1β levels, and GA with decreased levels of Eotaxin-1, MIP-1β, and ICAM-1. The detailed results of the generalized estimating equations are listed in Supplementary Table 3.

**Table 5:** Summary of the association between an infant's gestational age and comorbidities with changes in serum cytokine levels between PMA 34 weeks and PMA 38 weeks analyzed using generalized estimating equations.

Time/Infant factors	Cytokine increased	*P* value	Cytokine decreased	*P* value
PMA 34 weeks to PMA 36 weeks				
Late-onset sepsis	No associations found			
Severe IVH ≧ grade 3	IL-2	0.024		
	IL-7	0.001		
	IL-11	0.027		
Necrotizing enterocolitis			Eotaxin-1	0.030
			Eotaxin-2	0.034
			IL-2	0.013
			IL-16	0.001
			MIP-1β	<0.001
			PDGF-BB	0.015
Hemodynamically significant PDA	IL-11	0.023		
	PDGF-BB	0.029		
Bronchopulmonary dysplasia	No associations found			
Gestational age, weeks			ICAM-1	0.003
PMA 36 weeks to PMA 38 weeks				
Late-onset sepsis			Eotaxin-1	0.023
			IL-2	0.032
			IL-11	0.001
Severe IVH ≧ grade 3	No associations found			
Necrotizing enterocolitis			Eotaxin-1	0.016
			IL-2	0.018
			IL-16	0.029
			PDGF-BB	0.018
			TIMP-2	0.015
Hemodynamically significant PDA	IL-11	<0.001		
Bronchopulmonary dysplasia			MIP-1β	0.036
Gestational age, weeks			Eotaxin-1	0.016
			MIP-1β	0.007
			ICAM-1	0.002

## Discussion

The primary aim of this study was to identify potential biomarkers during a relatively stable period that could predict neurodevelopmental outcomes in preterm infants. Both single time-point cytokine levels and changes over specific intervals may serve as potential predictors. Most biomarkers identified during this phase were associated with favorable outcomes and showed good discriminatory ability, particularly changes in Eotaxin-2 from PMA 34 to 36 weeks (AUC 0.833) and PDGF-BB from PMA 34 to 38 weeks (AUC 0.821). In contrast, higher serum IFN-γ at PMA 34 weeks and greater increases in IL-17 from PMA 34 to 36 weeks were linked to adverse outcomes, with AUCs of 0.752 and 0.744. These findings highlight the potential clinical utility of cytokine profiling during PMA 34–38 weeks for identifying infants at risk of neurodevelopmental impairment.

### Biomarkers associated with detrimental effects

IFN-γ is a pro-inflammatory cytokine, and its role in neurodevelopment remains controversial. Some studies suggest that IFN-γ contributes to neuroinflammation and neurodegeneration in mouse models (15), with its absence enhancing cognitive performance through increased hippocampal plasticity (16). Conversely, other research has shown that IFN-γ can promote neurogenesis (17). IL-17, primarily secreted by T helper 17 (Th17) cells, is another inflammatory cytokine implicated in various chronic inflammatory neurological disorders (18). Lu et al. reviewed the role of neuroinflammation in neurological and psychiatric conditions and found IL-17 to be associated with several disorders, including autism spectrum disorder (ASD), Alzheimer's disease (AD), depression, and epilepsy (19). In one study, neonatal mice exposed to sevoflurane showed increased IL-17A expression in the hippocampus; deletion or inhibition of IL-17A attenuated sevoflurane-induced cognitive impairment by reducing hippocampal neuroinflammation (20). Although previous reviews have not reported associations between IFN-γ or IL-17 and neurodevelopmental outcomes in preterm infants (10, 21, 22), the elevated levels observed in our study may influence neurodevelopment based on their known physiological roles.

### Biomarkers with favorable effects consistent with previous findings

In our study, higher serum levels of IL-2, IL-7, IL-11, MIP-1α, MIP-1β, MIP-1δ, PDGF-BB, TIMP-2, and TNF-β were positively associated with better neurodevelopmental outcomes. These findings are consistent with previous research on these cytokines in other neurological disorders. IL-2 is known to regulate T-cell proliferation, particularly regulatory T cells (23, 24), thereby modulating inflammatory responses. In a mouse model of traumatic brain injury, IL-2 complex treatment was shown to alleviate inflammation and reduce blood-brain barrier disruption (25). Additionally, elevated levels of TNF-β and IL-2 have been reported at school age in children with a history of neonatal encephalopathy (26). IL-7 is a cytokine involved in B and T cell development (27), and plays a role in restoring immune function following illness (28). Although previous reviews have not established a direct link between IL-7 and neurodevelopment, IL-7 has demonstrated neurotrophic properties and may play an important role in neural development (29). IL-11, an anti-inflammatory cytokine in the IL-6 family (30), has shown neuroprotective effects in animal studies. In rat models, recombinant human IL-11 (rhIL-11) has been found to protect against cerebral ischemia-reperfusion injury (31) and exert anti-apoptotic effects in neonatal hypoxic-ischemic brain injury (32).

MIP-1α, MIP-1β, and MIP-1δ are chemokines that play essential roles in regulating leukocyte trafficking and modulating inflammatory responses (33, 34). MIP-1α, primarily secreted by macrophages, is crucial for recruiting inflammatory cells and has been implicated in various inflammatory diseases (35). In a study analyzing neonatal blood spot samples, lower levels of MIP-1α were associated with both ASD and developmental delays, while reduced MIP-1δ levels were specifically linked to developmental delays (36). However, another study assessing the inflammatory profiles of school-age children born extremely preterm (GA < 28 weeks) found no significant differences in MIP-1α or MIP-1β levels between children with motor, cognitive, or behavioral impairments and their unaffected peers (37).

PDGF-BB is a member of the platelet-derived growth factor (PDGF) family and is known for its roles in neuroprotection and cell survival (38). It has been shown to prevent neuronal apoptosis following ischemic neuronal injury (39, 40) and to promote the proliferation, differentiation, and migration of neuronal progenitor cells (41).

TIMPs are endogenous inhibitors of matrix metalloproteinases. Upregulation of TIMP-2 has been associated with neuronal proliferation and differentiation (42). Research also indicates that TIMP-2 can inhibit microglial activation, suggesting its neuroprotective potential. These findings suggest that TIMP-2 may be a therapeutic target for neuroinflammatory disorders (42). TNF is a family of proinflammatory cytokines involved in immune defense and capable of inducing cell death and neurodegeneration. While TNF-α has been associated with adverse neurodevelopmental outcomes (21), the role of TNF-β is less well documented. Nevertheless, TNF-β may exert protective effects (43) and has been found to be elevated at school age in children with a history of neonatal encephalopathy (26).

### Biomarkers with favorable effects different from previous findings

In our study, higher serum levels of Eotaxin-1, Eotaxin-2, ICAM-1, and IL-16 were observed in the non-NDI group, suggesting a potential protective effect on neurodevelopmental outcomes in preterm infants. However, these findings are not consistent with prior reports on these cytokines in the context of other neurological disorders. Eotaxins, part of the C-C motif chemokine family, are potent chemoattractants for eosinophils and play key roles in innate immunity. This group includes Eotaxin-1 [also known as C-C motif chemokine ligand (CCL)11], Eotaxin-2 (CCL24), and Eotaxin-3 (CCL26) (44). Eotaxin-1 and Eotaxin-2 are inflammatory chemokines involved in eosinophil recruitment and the Th2-mediated immune response (45). Elevated serum and cerebrospinal fluid levels of Eotaxin-1 and Eotaxin-2 have been reported in adults with various neurodegenerative diseases (44). ICAM-1 plays a central role in immune responses by facilitating leukocyte trafficking into inflamed tissues and regulating blood-brain barrier integrity (46, 47). Elevated intrathecal, but not serum, levels of soluble ICAM-1 have been reported in inflammatory neurological conditions such as viral meningoencephalitis and bacterial meningitis (46). Additionally, sustained elevation of inflammation-related proteins, including ICAM-1, during the first postnatal month in extremely preterm infants has been associated with increased risk of cognitive impairment at 10 years of age (48). IL-16 is a proinflammatory cytokine expressed in the brain under inflammatory conditions, as shown in rat models (49). Elevated cord blood IL-16 levels have also been linked to severely abnormal neurodevelopmental outcomes at three years of age in infants with perinatal asphyxia and hypoxic-ischemic encephalopathy (50).

We identified several biomarkers associated with either favorable or detrimental effects on the neurodevelopmental outcomes of preterm infants. Some biomarkers had not been previously reported in neonatal groups, and we referred to results from studies conducted in adults or animal models to interpret these findings. However, certain biomarkers with favorable effects were not mechanistically consistent. Although previous studies have linked Eotaxin-1, Eotaxin-2, ICAM-1, and IL-16 to neuroinflammation and adverse neurological outcomes in other populations, our findings suggest a potential protective role in preterm infants. This discrepancy may reflect developmental stage–specific or context-dependent functions of these cytokines. For instance, during early brain development, certain inflammatory mediators may support neurovascular maturation, immune regulation, or tissue remodeling rather than cause injury (51, 52).

Another critical point in explaining the association between these biomarkers and neurodevelopment is that the development of the human immune system and central nervous system is a continuous and dynamic process, characterized by various changes throughout infancy and childhood, and may vary among individuals due to different external stimuli (53, 54). For instance, extremely preterm infants born before 28 weeks, who have not undergone the third-trimester adaptation processes to tolerate maternal and self-antigens, exhibit different responses to inflammatory insults (55). Additionally, at different times after birth, varying cytokine responses to bacterial lipopolysaccharide stimulation were observed (56). This may explain why certain biomarkers previously reported to be significant at different postnatal time periods in other studies did not yield significant results in our research. Similar findings were reported in one review study, which demonstrated that biomarkers significantly associated with neurodevelopmental outcomes varied across different postnatal time periods (21). Furthermore, due to the different developmental statuses of the central nervous and immune systems across infancy, childhood, and adulthood, referencing studies conducted in adults or animal models to explain these associations may not always be suitable.

### Associations between preterm comorbidities and biomarkers

In our study, we also found that preterm comorbidities were associated with changes in cytokine levels between PMA 34 and 38 weeks, despite the fact that most of these conditions— such as NEC, HsPDA and severe IVH—typically develop during the first month of life. Indeed, several biomarkers have been reported to be associated with these comorbidities. Cytokines including IL-1β, IL-6, IL-8, IL-18, and TNF-α have been implicated in the pathophysiology of preterm IVH (57, 58). Higher serum levels of IL-6 on day 1 (59) and IL-8 on days 1, 7, and 14 after birth (60) have been linked to an increased risk of IVH. Similarly, elevated levels of inflammatory cytokines such as TNF-α, IL-1, IL-6, IL-8, IL-10, MCP-1/CCL2, and MIP-1α have been associated with the persistence of PDA (61, 62).

NEC is a well-recognized condition marked by a robust inflammatory response and is a significant risk factor for adverse neurodevelopmental outcomes. Infants with NEC exhibit elevated levels of inflammatory cytokines, including IL-1β, IL-6, IL-8, IL-10, MCP-1/CCL2, and MIP-1β/CCL3, along with decreased levels of anti-inflammatory cytokines such as TGF-β and IL-2 (63). Notably, approximately 40% of infants with NEC develop NDI (64), and the severity of intestinal injury has been shown to correlate with an increased risk of NDI (65). The pathogenesis of NDI in the context of NEC is believed to involve gut–brain axis interactions. Specifically, microbial dysbiosis and intestinal injury can trigger systemic inflammation and the release of pro-inflammatory cytokines such as TNF-α, IL-1β, and IL-6, which may cross the immature blood–brain barrier and contribute to neuroinflammation, ultimately disrupting normal brain development (66–68). Neonatal sepsis is another well-established risk factor for adverse neurodevelopmental outcomes (69–72). Potential pathogenic mechanisms include systemic inflammation, cytokine dysregulation, and subsequent white matter injury. Elevated serum levels of several pro-inflammatory cytokines—such as IL-6, IL-8, IL-10, IL-1β, TNF-α, and MCP-1—have been associated with neonatal sepsis (73–75). During the acute phase of sepsis, pro-inflammatory cytokines such as TNF-α and IL-6 predominate, whereas the post-acute phase is characterized by increased levels of anti-inflammatory cytokines, including IL-10 (74).

BPD is a common morbidity in preterm infants. Numerous serum biomarkers have been confirmed to be associated with the subsequent development of BPD (76). Higher levels of proinflammatory, profibrotic and angiogenic cytokines (IL-6, IL-8, IL-10, MCP-1) within the first 5 days after birth have been linked to the later development of moderate to severe BPD (77). Another study analyzed biomarkers before 21 days of age and found that significant biomarkers varied when measured at different time points (78). The study concluded that abnormalities in the transition from innate immune response to adaptive immune response may be related to the occurrence of BPD. Survivors with neonatal BPD are at a higher risk of developing NDI (79, 80). One possible explanation is that preterm infants are generally sicker and more prone to nutritional problems, which may disrupt brain development. Another potential reason is the frequent and prolonged periods of hypoxemia, which can directly cause brain injury (80). Studies investigating the association between BPD, cytokines, and NDI are limited. However, it is theoretically plausible that persistent systemic inflammatory reactions or the effects of inflammatory biomarkers could contribute to NDI. Further research is needed to better understand these relationships.

As discussed above, changes in biomarkers associated with preterm comorbidities have been linked to neurodevelopmental outcomes. However, most existing studies have focused on earlier life stages—primarily within the first month after birth or during the acute phase of illness. In contrast, our study explores the potential long-term effects of comorbidities on biomarkers during a later and more stable period—between 34 and 38 weeks PMA—to assess the relationships among comorbidities, biomarkers, and neurodevelopmental outcomes. Importantly, our analysis focused on identifying biomarkers during this period that may reflect the long-term impact of preterm complications, rather than assessing how a single disease alters biomarker levels. This individual-centered approach emphasizes which biomarkers are associated with neurodevelopmental outcomes and explores their connections to common preterm morbidities, providing new insights into how these conditions may influence brain development.

Another potential confounding factor affecting neurodevelopment is the use of antenatal magnesium sulfate (MgSO₄) and antenatal corticosteroid therapy (ACS). Antenatal MgSO4 is known to provide neuroprotection in preterm births (81–83). Proposed mechanisms include promoting hemodynamic stability, preventing excitotoxic injury, stabilizing neurons, and exerting antioxidant effects (84, 85). Additionally, its anti-inflammatory properties have been reported, helping to reduce proinflammatory cytokines such as IL-1β and TNF-α (86). ACS, administered for threatened preterm birth, accelerates fetal lung maturation and has been proven to reduce mortality, as well as the risk of RDS and IVH (87). However, its impact on long-term neurodevelopmental outcomes remains uncertain. A meta-analysis found that ACS reduced the risk of neurodevelopmental impairment only in extremely preterm births, whereas in late-preterm and full-term births, ACS was associated with an increased risk of neurocognitive disorders (88). Lower cord blood levels of IL-6 have been observed in very low birth weight preterm infants after ACS administration (89). However, its influence on cytokines associated with neurodevelopmental outcomes remains poorly understood. One study reported reduced cord blood levels of neurotrophin-3 (NT-3) in late-preterm infants who received ACS, which could potentially affect neuronal growth, differentiation, and survival (90). Conversely, another study found no significant differences in cord blood concentrations of pro-inflammatory, anti-inflammatory, or neurotrophic cytokines after ACS administration, despite trends toward attenuation of the inflammatory response (91). In our study, however, neither MgSO₄ nor ACS use was significantly associated with neurodevelopmental outcomes.

### Limitations

This study has several limitations. First, the relatively small sample size is a key limitation; although 50 cases were initially enrolled, only 35 were included in the final analysis. Although additional details regarding the reasons for exclusion have been provided, the potential selection bias resulting from the exclusion of 15 out of 50 infants (30%) remains a notable concern. Future studies with larger cohorts are needed to validate and extend these findings. Second, although the BSID-III assessment at 24 months of age is most predictive for neurodevelopment, the assessments were only available at 12 months of age due to the study timeline, as most participants had not yet completed the 24-month follow-up. Longitudinal data will be important to evaluate the long-term impact of these biomarkers on neurodevelopmental outcomes. As aforementioned, many studies have focused on cytokines measured at birth or during the early postnatal stage. Cytokines during this period provide valuable insights into the inflammatory processes in preterm infants. However, due to the limitations of our study design, measurements at birth or during the early postnatal period were not included, preventing us from comparing the differences and changes in cytokines between the early stage and the relatively stable later stage. Lastly, although we identified several biomarkers with significant associations, we were unable to fully elucidate the complex network through which these biomarkers influence neurodevelopment. Further research is needed to clarify the mechanistic roles of individual biomarkers and to explore their potential interactions.

## Conclusions

Our study suggests that biomarkers measured at PMA 34–38 weeks, including both absolute cytokine levels and their trajectories, may help identify preterm infants at risk for neurodevelopmental impairment. While most biomarkers were associated with favorable outcomes, IFN-γ and IL-17 correlated with adverse neurodevelopmental outcomes. All identified biomarkers demonstrated good discriminatory ability. Additionally, several common neonatal comorbidities significantly influenced cytokine levels, underscoring the complex interplay between systemic inflammation, clinical complications, and neurodevelopment.

## Data Availability

The original contributions presented in the study are included in the article/Supplementary Material, further inquiries can be directed to the corresponding author/s.
