# Supplementary material for: Serum cytokine profiles at near term-equivalent age and their association with neurodevelopmental outcomes in preterm infants: an exploratory study
Source: Front Pediatr. 2025 Aug 28;13:1667521. doi: 10.3389/fped.2025.1667521 (PMC12423045; doi:10.3389/fped.2025.1667521)
Supplement: Supplementary file 1 [file Table1.docx]

Supplementary Table 1. Cytokine levels at each time point between infants with and without neurodevelopmental impairment.

| Cytokines (pg/mL) | Non-NDI (N=29) | NDI (N=9) | *P* |
| --- | --- | --- | --- |
| **BLC** |  |  |  |
| PMA 34 weeks | 8.53 (1.31, 26.96) | 11.83 (4.24, 31.02) | 0.342 |
| PMA 36 weeks | 8.40 (0.82, 29.36) | 7.20 (2.82, 35.75) | 0.670 |
| PMA 38 weeks | 8.81 (2.19, 47.95) | 7.57 (2.03, 56.72) | 0.781 |
| **Eotaxin-1** |  |  |  |
| PMA 34 weeks | 346.02 (21.15, 686.08) | 337.47 (67.61, 1151.50) | 0.810 |
| PMA 36 weeks | 361.42 (60.66, 588.72) | 152.09 (76.02, 746.34) | 0.061 |
| PMA 38 weeks | 342.69 (75.06, 846.85) | 277.02 (115.77, 562.20) | 0.362 |
| **Eotaxin-2** |  |  |  |
| PMA 34 weeks | 301.47 (33.10, 515.85) | 325.99 (46.13, 690.73) | 0.697 |
| PMA 36 weeks | 336.28 (52.43, 624.08) | 130.49 (58.34, 388.64) | 0.023 |
| PMA 38 weeks | 322.28 (81.82, 669.78) | 191.54 (136.15, 437.53) | 0.224 |
| **G-CSF** |  |  |  |
| PMA 34 weeks | 19.28 (0.00, 54.26) | 9.73 (1.44, 914.9) | 0.868 |
| PMA 36 weeks | 7.70 (0.00, 55.31) | 11.67 (0.00, 40.01) | 0.810 |
| PMA 38 weeks | 1.00 (0.00, 3345.74) | 7.31 (0.00, 94.09) | 0.670 |
| **GM-CSF** |  |  |  |
| PMA 34 weeks | 60.03 (0.00, 141.20) | 58.47 (0.00, 139.69) | 0.956 |
| PMA 36 weeks | 45.69 (0.00, 210.46) | 28.76 (0.00, 138.94) | 0.224 |
| PMA 38 weeks | 82.62 (7.02, 354.66) | 31.63 (0.00, 222.57) | 0.101 |
| **I-309** |  |  |  |
| PMA 34 weeks | 1.09 (0.00, 23.90) | 0.00 (0.00, 100.88) | 0.926 |
| PMA 36 weeks | 0.00 (0.00, 43.84) | 0.00 (0.00, 88.76) | 0.382 |
| PMA 38 weeks | 0.00 (0.00, 883.38) | 0.00 (0.00, 118.9) | 0.516 |
| **ICAM-1** |  |  |  |
| PMA 34 weeks | 3267.71 (1531.04, 4224.16) | 3190.84 (2426.97, 3607.09) | 0.897 |
| PMA 36 weeks | 3297.51 (1150.75, 4441.53) | 2827.63 (2194.42, 3753.87) | 0.093 |
| PMA 38 weeks | 3202.60 (1830.19, 3867.24) | 2622.08 (1722.03, 3682.77) | 0.093 |
| **IFN-γ** |  |  |  |
| PMA 34 weeks | 1.59 (0.00, 6.48) | 4.44 (0.42, 9.04) | 0.025 |
| PMA 36 weeks | 1.28 (0.00, 78.62) | 2.15 (0.00, 98.75) | 0.255 |
| PMA 38 weeks | 2.06 (0.00, 331.09) | 2.17 (0.00, 166.73) | 0.565 |
| **IL-1α** |  |  |  |
| PMA 34 weeks | 0.00 (0.00, 57.29) | 0.24 (0.00, 57.84) | 0.197 |
| PMA 36 weeks | 0.00 (0.00, 30.79) | 0.00 (0.00, 15.92) | 0.469 |
| PMA 38 weeks | 0.00 (0.00, 87.61) | 0.00 (0.00, 26.14) | 0.810 |
| **IL-1β** |  |  |  |
| PMA 34 weeks | 0.69 (0.00, 15.28) | 2.44 (0.00, 6.35) | 0.197 |
| PMA 36 weeks | 0.43 (0.00, 9.07) | 1.36 (0.00, 9.52) | 0.697 |
| PMA 38 weeks | 2.02 (0.00, 26.07) | 0.00 (0.00, 17.37) | 0.492 |
| **IL-1Rα** |  |  |  |
| PMA 34 weeks | 2.66 (0.00, 10.38) | 1.10 (0.00, 24.77) | 0.781 |
| PMA 36 weeks | 1.86 (0.00, 5.24) | 0.72 (0.00, 82.07) | 1.000 |
| PMA 38 weeks | 1.92 (0.00, 623.21) | 1.16 (0.00, 27.75) | 0.985 |
| **IL-2** |  |  |  |
| PMA 34 weeks | 19.11 (0.12, 72.41) | 13.82 (0.00, 54.36) | 0.516 |
| PMA 36 weeks | 19.90 (4.03, 46.06) | 8.28 (0.00, 29.98) | 0.086 |
| PMA 38 weeks | 21.49 (5.19, 140.18) | 14.04 (1.44, 22.42) | 0.042 |
| **IL-4** |  |  |  |
| PMA 34 weeks | 2.32 (0.00, 69.25) | 2.67 (0.00, 14.58) | 0.810 |
| PMA 36 weeks | 1.71 (0.00, 74.99) | 4.24 (0.00, 27.94) | 0.342 |
| PMA 38 weeks | 9.95 (0.00, 46.24) | 2.95 (0.00, 48.64) | 0.810 |
| **IL-5** |  |  |  |
| PMA 34 weeks | 10.11 (2.31, 36.3) | 12.21 (4.65, 35.14) | 0.540 |
| PMA 36 weeks | 8.96 (0.00, 73.78) | 13.21 (0.00, 92.91) | 0.868 |
| PMA 38 weeks | 12.11 (0.69, 100.23) | 12.11 (0.00, 189.72) | 0.810 |
| **IL-6** |  |  |  |
| PMA 34 weeks | 6.71 (0.00, 75.35) | 9.53 (3.70, 20.84) | 0.469 |
| PMA 36 weeks | 6.47 (1.78, 147.95) | 6.59 (0.00, 25.21) | 0.516 |
| PMA 38 weeks | 9.07 (1.07, 2374.81) | 4.77 (1.10, 65.95) | 0.056 |
| **IL-6R** |  |  |  |
| PMA 34 weeks | 5091.66 (3859.11, 5958.45) | 4725.58 (4170.19, 5909.33) | 0.725 |
| PMA 36 weeks | 5022.69 (3832.31, 5850.21) | 4876.30 (3785.49, 5836.49) | 0.516 |
| PMA 38 weeks | 4954.66 (2040.36, 5962.78) | 5002.48 (3216.82, 5642.88) | 0.985 |
| **IL-7** |  |  |  |
| PMA 34 weeks | 55.64 (0.49, 175.03) | 61.93 (22.59, 174.68) | 0.305 |
| PMA 36 weeks | 63.78 (0.00, 354.97) | 28.12 (0.00, 111.09) | 0.138 |
| PMA 38 weeks | 93.62 (0.00, 296.84) | 52.57 (0.00, 325.55) | 0.093 |
| **IL-8** |  |  |  |
| PMA 34 weeks | 8.17 (0.81, 44.03) | 12.06 (5.29, 49.73) | 0.305 |
| PMA 36 weeks | 6.20 (1.29, 87.68) | 5.21 (0.82, 37.20) | 0.323 |
| PMA 38 weeks | 6.49 (0.49, 345.40) | 5.60 (0.00, 108.25) | 0.342 |
| **IL-10** |  |  |  |
| PMA 34 weeks | 25.03 (0.25, 147.85) | 25.17 (6.58, 68.70) | 0.590 |
| PMA 36 weeks | 21.92 (0.00, 179.38) | 17.65 (6.82, 36.27) | 0.697 |
| PMA 38 weeks | 29.20 (5.05, 174.26) | 18.95 (3.16, 59.05) | 0.110 |
| **IL-11** |  |  |  |
| PMA 34 weeks | 96.67 (0.00, 374.27) | 75.01 (0.00, 192.15) | 0.670 |
| PMA 36 weeks | 62.04 (0.00, 756.31) | 59.56 (0.00, 164.19) | 0.725 |
| PMA 38 weeks | 86.40 (0.00, 543.21) | 17.82 (0.00, 187.47) | 0.028 |
| **IL-12p40** |  |  |  |
| PMA 34 weeks | 13.41 (0.00, 58.35) | 9.67 (6.29, 50.12) | 0.590 |
| PMA 36 weeks | 17.33 (0.56, 41.97) | 10.54 (1.57, 43.96) | 0.171 |
| PMA 38 weeks | 20.94 (4.31, 530.49) | 12.81 (4.71, 59.31) | 0.224 |
| **IL-12p70** |  |  |  |
| PMA 34 weeks | 0.31 (0.00, 2.08) | 0.20 (0.00, 0.86) | 1.000 |
| PMA 36 weeks | 0.11 (0.00, 1.86) | 0.03 (0.00, 0.75) | 0.616 |
| PMA 38 weeks | 0.40 (0.00, 2.25) | 0.11 (0.00, 0.93) | 0.305 |
| **IL-13** |  |  |  |
| PMA 34 weeks | 0.90 (0.00, 2.87) | 1.22 (0.00, 1.78) | 0.753 |
| PMA 36 weeks | 0.55 (0.00, 6.28) | 0.88 (0.00, 8.16) | 0.781 |
| PMA 38 weeks | 0.96 (0.00, 6.80) | 0.86 (0.00, 16.29) | 0.540 |
| **IL-15** |  |  |  |
| PMA 34 weeks | 1.09 (0.00, 5.25) | 1.47 (0.61, 6.11) | 0.101 |
| PMA 36 weeks | 0.82 (0.00, 50.45) | 0.77 (0.02, 51.23) | 0.255 |
| PMA 38 weeks | 1.22 (0.00, 57.14) | 1.57 (0.45, 138.43) | 0.403 |
| **IL-16** |  |  |  |
| PMA 34 weeks | 189.92 (5.98, 1099.50) | 509.29 (45.53, 1142.25) | 0.239 |
| PMA 36 weeks | 389.70 (13.82, 1023.00) | 50.82 (8.40, 409.72) | 0.034 |
| PMA 38 weeks | 350.62 (12.17, 1179.41) | 56.13 (11.32, 965.40) | 0.079 |
| **IL-17** |  |  |  |
| PMA 34 weeks | 3.58 (0.00, 49.73) | 2.86 (0.00, 9.06) | 0.643 |
| PMA 36 weeks | 1.81 (0.00, 47.01) | 5.56 (0.00, 27.73) | 0.067 |
| PMA 38 weeks | 3.66 (0.00, 73.60) | 6.48 (0.00, 41.54) | 0.897 |
| **MCP-1** |  |  |  |
| PMA 34 weeks | 284.54 (57.44, 550.76) | 222.83 (147.03, 607.25) | 0.810 |
| PMA 36 weeks | 202.21 (106.66, 523.73) | 192.88 (123.59, 424.55) | 0.868 |
| PMA 38 weeks | 199.99 (93.76, 675.17) | 231.42 (108.22, 435.53)) | 0.643 |
| **MCSF** |  |  |  |
| PMA 34 weeks | 0.01 (0.00, 11.20) | 0.70 (0.00, 15.65) | 0.540 |
| PMA 36 weeks | 0.13 (0.00, 5.26) | 0.00 (0.00, 0.97) | 0.616 |
| PMA 38 weeks | 0.28 (0.00, 38.02) | 0.10 (0.00, 8.59) | 0.670 |
| **MIG** |  |  |  |
| PMA 34 weeks | 67.06 (0.00, 223.25) | 65.90 (0.00, 538.41) | 0.670 |
| PMA 36 weeks | 40.29 (0.64, 349.92) | 28.23 (0.00, 233.37) | 0.926 |
| PMA 38 weeks | 72.52 (11.38, 7110.99) | 51.82 (0.00, 527.16) | 0.643 |
| **MIP-1α** |  |  |  |
| PMA 34 weeks | 68.38 (12.76, 224.29) | 130.41 (52.35, 365.39) | 0.051 |
| PMA 36 weeks | 67.91 (18.81, 438.39) | 66.73 (32.25, 143.85) | 0.868 |
| PMA 38 weeks | 78.51 (10.55, 1060.03) | 72.86 (32.41, 256.25) | 0.810 |
| **MIP-1β** |  |  |  |
| PMA 34 weeks | 27.84 (2.89, 81.44) | 62.74 (16.10, 115.37) | 0.056 |
| PMA 36 weeks | 26.12 (5.19, 61.05) | 17.16 (13.57, 72.67) | 0.362 |
| PMA 38 weeks | 27.69 (3.06, 234.15) | 21.70 (13.56, 70.88) | 0.753 |
| **MIP-1δ** |  |  |  |
| PMA 34 weeks | 301.27 (187.43, 496.58) | 256.74 (169.40, 302.41) | 0.079 |
| PMA 36 weeks | 282.19 (154.84, 455.66) | 262.04 (112.50, 344.14) | 0.073 |
| PMA 38 weeks | 316.77 (143.57, 474.35) | 246.99 (147.89, 295.22) | 0.018 |
| **PDGF-BB** |  |  |  |
| PMA 34 weeks | 17393.53 (1472.78, 22385.23) | 17210.16 (4235.79, 24451.61) | 0.643 |
| PMA 36 weeks | 17368.56 (6700.26, 23205.80) | 11014.52 (2059.40, 20522.66) | 0.025 |
| PMA 38 weeks | 17843.52 (8301.64, 24273.24) | 15539.39 (3049.97, 22588.55) | 0.119 |
| **RANTES** |  |  |  |
| PMA 34 weeks | 5944.91 (3091.56, 7704.52) | 5030.16 (3953.80, 6561.46) | 0.446 |
| PMA 36 weeks | 6144.78 (3846.91, 7023.17) | 5848.15 (3893.74, 6528.69) | 0.540 |
| PMA 38 weeks | 5992.15 (3720.80, 7034.37) | 5760.41 (3811.63, 7156.20) | 0.565 |
| **TIMP-1** |  |  |  |
| PMA 34 weeks | 4051.47 (3655.78, 5226.58) | 4134.68 (3877.16, 4748.80) | 0.516 |
| PMA 36 weeks | 4150.32 (3495.16, 5597.28) | 4086.97 (3651.47, 4709.39) | 0.985 |
| PMA 38 weeks | 4166.98 (3300.60, 5380.28) | 4208.66 (3759.18, 5004.51) | 0.725 |
| **TIMP-2** |  |  |  |
| PMA 34 weeks | 5079.46 (2130.64, 6519.09) | 4732.85 (3828.42, 6505.92) | 0.670 |
| PMA 36 weeks | 5456.47 (3771.14, 6688.59) | 4124.53 (3593.22, 5773.04) | 0.051 |
| PMA 38 weeks | 5558.79 (3491.90, 6786.18) | 4675.14 (3766.40, 5882.53) | 0.042 |
| **TNF-α** |  |  |  |
| PMA 34 weeks | 3.02 (0.00, 10.95) | 4.02 (0.12, 7.84) | 0.403 |
| PMA 36 weeks | 2.57 (0.01, 19.74) | 3.35 (0.00, 13.18) | 0.670 |
| PMA 38 weeks | 3.42 (0.00, 36.13) | 3.15 (0.00, 38.91) | 0.446 |
| **TNF-β** |  |  |  |
| PMA 34 weeks | 452.87 (13.22, 1385.33) | 451.39 (0.00, 1026.14) | 0.616 |
| PMA 36 weeks | 398.10 (0.00, 1087.44) | 142.41 (0.00, 550.87) | 0.031 |
| PMA 38 weeks | 402.58 (57.76, 2003.58) | 303.76 (74.85, 1162.25) | 0.382 |
| **TNF R1** |  |  |  |
| PMA 34 weeks | 7457.69 (4746.40, 9158.48) | 7030.97 (5066.86, 9556.22) | 0.382 |
| PMA 36 weeks | 7437.17 (5165.08, 8756.54) | 6051.94 (5336.05, 9457.79) | 0.061 |
| PMA 38 weeks | 7004.12 (4503.33, 10143.04) | 6058.74 (5273.00, 9062.07) | 0.073 |
| **TNF R2** |  |  |  |
| PMA 34 weeks | 7331.66 (4062.31, 9618.53) | 7113.111 (5778.02, 10031.99) | 0.725 |
| PMA 36 weeks | 7360.17 (4435.57, 9450.97) | 6626.40 (5081.63, 10184.15) | 0.184 |
| PMA 38 weeks | 7016.81 (3208.97, 10978.49) | 6009.19 (3871.68, 10018.9) | 0.079 |

Supplementary Table 2.Changes in cytokine levels at each time interval between infants with and without neurodevelopmental impairment.

| Cytokine changes (pg/mL) | Non-NDI (N=29) | NDI (N=9) | *P* |
| --- | --- | --- | --- |
| **BLC** |  |  |  |
| PMA 34-36 weeks | -0.50 (-11.91, 10.48) | -2.71 (-21.94, 27.64) | 0.184 |
| PMA 36-38 weeks | 1.84 (-8.86, 36.96) | -0.19 (-2.84, 20.97) | 0.838 |
| PMA 34-38 weeks | 0.59 (-14.84, 44.35) | -2.84 (-16.14, 48.61) | 0.149 |
| **Eotaxin-1** |  |  |  |
| PMA 34-36 weeks | 38.65 (-213.34, 334.51) | -119.21 (-409.37, 74.67) | 0.009 |
| PMA 36-38 weeks | -14.28 (-254.04, 341.17) | 30.37 (-184.13, 214.3) | 0.362 |
| PMA 34-38 weeks | 11.97 (-468.38, 371.14) | -60.46 (-589.30, 48.16) | 0.056 |
| **Eotaxin, 2** |  |  |  |
| PMA 34-36 weeks | 55.26 (-221.93, 291.60) | -101.61 (-437.87, 19.79) | 0.002 |
| PMA 36-38 weeks | 1.00 (-172.19, 340.09) | 26.74 (-120.04, 347.29) | 0.305 |
| PMA 34-38 weeks | 19.58 (-181.32, 578.05) | -90.58 (-477.94, 95.24) | 0.079 |
| **G-CSF** |  |  |  |
| PMA 34-36 weeks | -7.46 (-45.98, 35.57) | 5.53 (-18.8, 54.08) | 0.897 |
| PMA 36-38 weeks | 0.15 (-45.14, 3337.47) | 5.53 (-18.8, 54.08) | 0.616 |
| PMA 34-38 weeks | -6.97 (-40.06, 3291.48) | -5.83 (-914.9, 88.85) | 0.897 |
| **GM-CSF** |  |  |  |
| PMA 34-36 weeks | 8.99 (-106.79, 121.96) | -31.14 (-60.32, 31.45) | 0.128 |
| PMA 36-38 weeks | 13.82 (-63.92, 343.97) | 3.66 (-50.71, 179.23) | 0.926 |
| PMA 34-38 weeks | 16.42 (-68.01, 287.43) | -20.05 (-51.45, 132.45) | 0.184 |
| **I-309** |  |  |  |
| PMA 34-36 weeks | 0.00 (-23.90, 32.37) | 0.00 (-100.88, 19.75) | 0.810 |
| PMA 36-38 weeks | 0.00 (-13.49, 883.38) | 0.00 (-4.33, 110.49) | 0.670 |
| PMA 34-38 weeks | 0.00 (-23.90, 883.38) | 0.00 (-100.88, 118.90) | 0.565 |
| **ICAM-1** |  |  |  |
| PMA 34-36 weeks | -17.02 (-962.25, 1357.59) | -252.85 (-1236.46, 300.49) | 0.119 |
| PMA 36-38 weeks | -67.34 (-1058.45, 1718.56) | -137.64 (-1005.44, 427.65) | 0.781 |
| PMA 34-38 weeks | -69.09 (-1069.27, 756.30) | -399.92 (-808.81, 151.82) | 0.010 |
| **IFN-γ** |  |  |  |
| PMA 34-36 weeks | -0.04 (-3.72, 72.14) | -0.13 (-7.98, 98.33) | 0.956 |
| PMA 36-38 weeks | 0.53 (-4.19, 328.86) | 0.19 (-0.98,, 67.98) | 0.781 |
| PMA 34-38 weeks | 0.20 (-3.99, 326.94) | 0.05 (-6.87, 166.32) | 0.492 |
| **IL-1α** |  |  |  |
| PMA 34-36 weeks | 0.00 (-57.29, 30.79) | 0.00 (-57.84, 8.16) | 0.838 |
| PMA 36-38 weeks | 0.00 (-30.79, 75.62) | 0.00 (-8.57, 21.89) | 0.540 |
| PMA 34-38 weeks | 0.00 (-13.13, 87.61) | 0.00 (-57.84, 26.14) | 0.224 |
| **IL-1β** |  |  |  |
| PMA 34-36 weeks | 0.00 (-13.12, 5.63) | -0.90 (-6.35, 8.11) | 0.403 |
| PMA 36-38 weeks | 0.95 (-7.36, 25.67) | 0.00 (-2.26, 7.84) | 0.590 |
| PMA 34-38 weeks | 0.00 (-15.28, 26.07) | -0.90 (-6.35, 15.95) | 0.540 |
| **IL-1Rα** |  |  |  |
| PMA 34-36 weeks | 0.00 (-8.29, 1.64) | 0.00 (-3.29, 57.30) | 0.362 |
| PMA 36-38 weeks | 0.04 (-5.12, 618.57) | 0.00 (-62.36, 20.80) | 0.697 |
| PMA 34-38 weeks | 0.00 (-5.45, 620.21) | 0.00 (-5.06, 25.18) | 0.868 |
| **IL-2** |  |  |  |
| PMA 34-36 weeks | -0.83 (-41.77, 39.05) | -3.58 (-24.38, 7.41) | 0.403 |
| PMA 36-38 weeks | 2.89 (-18.48, 126.92) | 2.09 (-8.97, 14.04) | 0.897 |
| PMA 34-38 weeks | 3.64 (-48.18, 109.00) | -1.56 (-33.15, 14.04) | 0.224 |
| **IL-4** |  |  |  |
| PMA 34-36 weeks | 0.00 (-63.43, 69.61) | 0.00 (-12.83, 27.94) | 0.540 |
| PMA 36-38 weeks | 2.96 (-66.09, 46.24) | 0.35 (-13.23, 20.69) | 0.540 |
| PMA 34-38 weeks | 3.88 (-69.25, 33.43) | 1.53 (-14.58, 48.64) | 0.810 |
| **IL-5** |  |  |  |
| PMA 34-36 weeks | -0.93 (-25.40, 58.66) | -1.39 (-20.51, 78.51) | 0.781 |
| PMA 36-38 weeks | 4.87 (-9.87, 75.05) | 0.00 (-6.04, 96.81) | 0.781 |
| PMA 34-38 weeks | 2.64 (-28.15, 85.11) | 0.46 (-26.55, 175.33) | 0.725 |
| **IL-6** |  |  |  |
| PMA 34-36 weeks | -1.30 (-64.41, 130.07) | -3.78 (-14.25, 14.17) | 0.323 |
| PMA 36-38 weeks | 2.60 (-6.01, 2369.13) | -0.64 (-2.96, 40.74) | 0.210 |
| PMA 34-38 weeks | 0.05 (-14.58, 2373.15) | -2.68 (-16.83, 54.91) | 0.271 |
| **IL-6R** |  |  |  |
| PMA 34-36 weeks | -29.37 (-1730.56, 1446.16) | 44.84 (-1822.94, 559.88) | 0.926 |
| PMA 36-38 weeks | -104.87 (-2921.81, 798.94) | 24.94 (-1108.35, 852.32) | 0.590 |
| PMA 34-38 weeks | -124.94 (-2700.76, 766,21) | 36.94 (-1421.58, 860.13) | 0.810 |
| **IL-7** |  |  |  |
| PMA 34-36 weeks | 20.59 (-147.68, 301.48) | -23.40 (-126.01, 2.40) | 0.020 |
| PMA 36-38 weeks | 15.97 (-108.42, 158.79) | 5.08 (-54.80, 240.85) | 0.956 |
| PMA 34-38 weeks | 19.71 (-88.17, 243.35) | -23.01 (-93.00, 171.33) | 0.046 |
| **IL-8** |  |  |  |
| PMA 34-36 weeks | -3.33 (-36.35, 73.27) | -5.20 (-48.90, 24.77) | 0.342 |
| PMA 36-38 weeks | 0.65 (-11.11, 320.76) | 0.51 (-2.80, 71.06) | 0.868 |
| PMA 34-38 weeks | -1.19 (-29.67, 307.14) | -4.69 (-49.73, 95.82) | 0.239 |
| **IL-10** |  |  |  |
| PMA 34-36 weeks | -2.00 (-62.46, 31.70) | -5.04 (-60.49, 11.03) | 0.469 |
| PMA 36-38 weeks | 8.41 (-74.67, 152.85) | -7.20 (-14.08, 38.82) | 0.197 |
| PMA 34-38 weeks | 2.35 (-58.5, 163.48) | -6.45 (-47.55, 16.54) | 0.086 |
| **IL-11** |  |  |  |
| PMA 34-36 weeks | -26.70 (-374.27, 765.31) | -29.88 (-142.22, 69.48) | 0.868 |
| PMA 36-38 weeks | 38.53 (-765.31, 543.21) | 0.00 (-131.01, 48.69) | 0.119 |
| PMA 34-38 weeks | 9.05 (-359.75, 533.77) | -30.85 (-192.15, 49.39) | 0.197 |
| **IL-12p40** |  |  |  |
| PMA 34-36 weeks | 1.67 (-18.99, 28.48) | -1.61 (-26.19, 24.41) | 0.210 |
| PMA 36-38 weeks | 3.11 (-20.90, 518.95) | 3.19 (-21.02, 34.08) | 0.810 |
| PMA 34-38 weeks | 5.11 (-16.26, 499.95) | 3.40 (-22.99, 24.59) | 0.516 |
| **IL-12p70** |  |  |  |
| PMA 34-36 weeks | 0.00 (-1.70, 1.42) | -0.06 (-0.64, 0.03) | 0.725 |
| PMA 36-38 weeks | 0.06 (-1.41, 2.25) | 0.00 (-0.29, 0.35) | 0.590 |
| PMA 34-38 weeks | 0.00 (-2.08, 1.81) | -0.13 (-0.39, 0.10) | 0.403 |
| **IL-13** |  |  |  |
| PMA 34-36 weeks | -0.20 (-2.36, 5.70) | -0.18 (-1.00, 6.95) | 0.838 |
| PMA 36-38 weeks | 0.21 (-1.97, 3.27) | 0.00 (-0.21, 8.12) | 0.956 |
| PMA 34-38 weeks | 0.05 (-2.55, 6.22) | -0.31 (-1.00, 15.07) | 0.956 |
| **IL-15** |  |  |  |
| PMA 34-36 weeks | 0.00 (-2.44, 45.77) | -0.70 (-3.34, 50.01) | 0.516 |
| PMA 36-38 weeks | 0.41 (-2.27, 6.68) | 0.42 (-1.66, 87.20) | 0.753 |
| PMA 34-38 weeks | 0.08 (-2.67, 52.46) | -0.20 (-2.82, 137.21) | 0.697 |
| **IL-16** |  |  |  |
| PMA 34-36 weeks | 13.12 (-746.42, 991.24) | -114.10 (-1098.75, 124.71) | 0.038 |
| PMA 36-38 weeks | -4.00 (-604.31, 1133.38) | 5.31 (-78.58, 555.68) | 0.565 |
| PMA 34-38 weeks | 60.18 (-1024.98, 1106.11) | -181.60 (-1076.70, 680.39) | 0.056 |
| **IL-17** |  |  |  |
| PMA 34-36 weeks | -0.54 (-49.62, 44.58) | 4.30 (-6.39, 21.55) | 0.031 |
| PMA 36-38 weeks | 0.73 (-44.26, 73.60) | -0.74 (-11.12, 23.66) | 0.255 |
| PMA 34-38 weeks | 0.54 (-49.73, 67.63) | 0.00 (-2.86, 33.83) | 0.956 |
| **MCP-1** |  |  |  |
| PMA 34-36 weeks | -33.44 (-277.10, 146.48) | -55.77 (-327.26, 262.11) | 0.985 |
| PMA 36-38 weeks | -39.23 (-290.69, 369.11) | -20.21 (-79.29, 59.14) | 0.643 |
| PMA 34-38 weeks | -83.60 (-317.72, 383.22) | -67.94 (-322.82, 273.10) | 0.926 |
| **MCSF** |  |  |  |
| PMA 34-36 weeks | 0.00 (-11.20, 4.22) | 0.00 (-15.65, 0.06) | 0.255 |
| PMA 36-38 weeks | 0.00 (-5.26, 38.02) | 0.00 (-0.84, 7.83) | 0.956 |
| PMA 34-38 weeks | 0.00 (-8.71, 38.02) | 0.00 (-15.65, 7.90) | 0.926 |
| **MIG** |  |  |  |
| PMA 34-36 weeks | -5.79 (-195.95, 184.72) | 9.83 (-352.12, 143.07) | 0.382 |
| PMA 36-38 weeks | 10.38 (-194.02, 6997.12) | 0.00 (-180.64, 340.87) | 0.697 |
| PMA 34-38 weeks | 1.42 (-185.49, 6962.44) | -7.41 (-75.75, 100.57) | 0.697 |
| **MIP-1α** |  |  |  |
| PMA 34-36 weeks | 2.64 (-143.82, 267.24) | -36.32 (-329.90, 13.44) | 0.038 |
| PMA 36-38 weeks | 7.53 (-83.80, 979.56) | 4.65 (-38.12, 212.40) | 0.697 |
| PMA 34-38 weeks | 10.11 (-90.15, 835.74) | -50.30 (-292.52, 225.84) | 0.031 |
| **MIP-1β** |  |  |  |
| PMA 34-36 weeks | -4.13 (-55.47, 21.14) | -17.60 (-101.79, 1.06) | 0.007 |
| PMA 36-38 weeks | 1.05 (-17.78, 194.45) | 3.06 (-15.56, 49.53) | 0.810 |
| PMA 34-38 weeks | 3.37 (-47.12, 172.56) | -16.68 (-82.45, 7.26) | 0.011 |
| **MIP-1δ** |  |  |  |
| PMA 34-36 weeks | 6.34 (-133.37, 116.15) | 7.85 (-176.37, 50.07) | 0.985 |
| PMA 36-38 weeks | 10.71 (-138.35, 125.48) | 19.60 (-48.91, 48.44) | 0.643 |
| PMA 34-38 weeks | 10.43 (-144.63, 152.63) | 2.72 (-140.99, 69.67) | 0.753 |
| **PDGF-BB** |  |  |  |
| PMA 34-36 weeks | 1684.09 (-10464.61, 13902.01) | -351149 (-11691.39, 754.14) | 0.004 |
| PMA 36-38 weeks | 702.33 (-6360.10, 10301.67) | 1568.61 (-3308.42, 14759.51) | 0.446 |
| PMA 34-38 weeks | 1306.09 (-8018.05, 12897.32) | -3028.39 (-10927.76, 14054.45) | 0.056 |
| **RANTES** |  |  |  |
| PMA 34-36 weeks | 130.53 (-866.60, 1584.95) | -43.18 (-670.04, 1241.66) | 0.897 |
| PMA 36-38 weeks | -155.25 (-1660.83, 1930.59) | -117.75 (-768.28, 1070.99) | 0.516 |
| PMA 34-38 weeks | 79.66 (-1753.13, 1561.27) | -55.99 (-370.79, 1888.99) | 0.781 |
| **TIMP-1** |  |  |  |
| PMA 34-36 weeks | 40.14 (-734.97, 986.92) | 97.72 (-1097.33, 574.71) | 0.868 |
| PMA 36-38 weeks | -5.91 (-748.17, 553.81) | 96.40 (-731.55, 827.95) | 0.697 |
| PMA 34-38 weeks | 41.84 (-1249.24, 769.92) | -47.10 (-269.38, 654.24) | 0.838 |
| **TIMP-2** |  |  |  |
| PMA 34-36 weeks | 90.80 (-743.86, 3959.80) | -383.73 (-1050.79, 1040.19) | 0.042 |
| PMA 36-38 weeks | 232.24 (-2332.42, 2859.74) | 173.18 (-174.05, 737.77) | 0.926 |
| PMA 34-38 weeks | 413.94 (-1332.68, 4330.47) | -126.46 (-1196.50, 898.53) | 0.239 |
| **TNF-α** |  |  |  |
| PMA 34-36 weeks | 0.41 (-9.20, 15.89) | -0.29 (-3.66, 6.21) | 0.210 |
| PMA 36-38 weeks | 1.11 (-6.15, 16.40) | 0.44 (-2.47, 25.73) | 0.956 |
| PMA 34-38 weeks | 1.22 (-8.01, 32.29) | -0.12 (-4.32, 31.93) | 0.184 |
| **TNF-β** |  |  |  |
| PMA 34-36 weeks | -87.73 (-800.96, 1037.09) | -148.04 (-532.82, 103.09) | 0.305 |
| PMA 36-38 weeks | 55.09 (-573.71, 1729.45) | 196.26 (-124.21, 762.35) | 0.305 |
| PMA 34-38 weeks | -30.28 (-742.69, 1579.48) | -21.63 (-336.56, 380.37) | 0.956 |
| **TNF R1** |  |  |  |
| PMA 34-36 weeks | -367.61 (-2597.57, 2957.25) | -98.43 (-2313.65, 297.36) | 0.810 |
| PMA 36-38 weeks | 73.99 (-2597.82, 2162.18) | -221.05 (-1445.16, 698.87) | 0.565 |
| PMA 34-38 weeks | -574.90 (-3360.19, 2478.51) | -972.23 (-2381.87, 996.22) | 0.540 |
| **TNF R2** |  |  |  |
| PMA 34-36 weeks | -255.41 (-1945.73, 3408.58) | 152.17 (-3045.13, 708.32) | 0.926 |
| PMA 36-38 weeks | -155.43 (-3529.74, 2076.56) | -904.27 (-3741.32, 1047.04) | 0.255 |
| PMA 34-38 weeks | -397.65 (-4339.16, 2606.54) | -897.69 (-3668.77, 232.31) | 0.101 |

Supplementary Table 3. Infant's gestational age and comorbidities associated with changes in serum cytokines between PMA 34 weeks and PMA 38 weeks according to generalized estimating equations.

|  |  | **95% Wald confidence interval** | |  |
| --- | --- | --- | --- | --- |
| **Cytokines/Infant factors** | **Estimate** | **Lower limit** | **Upper limit** | **P value** |
| **PMA 34 weeks to 36 weeks** | | | | |
| **IFN-γ** |  |  |  |  |
| Late-onset sepsis | 2.652 | -10.553 | 15.857 | 0.694 |
| Severe IVH ≧ Grade 3 | 12.583 | -13.599 | 38.766 | 0.346 |
| Necrotizing enterocolitis | -9.714 | -32.365 | 12.937 | 0.401 |
| Hemodynamically significant PDA | -0.163 | -2.585 | 2.259 | 0.895 |
| Bronchopulmonary dysplasia | -12.945 | -35.389 | 9.498 | 0.258 |
| Gestational age, weeks | 0.488 | -0.358 | 1.334 | 0.258 |
| **IL-17** |  |  |  |  |
| Late-onset sepsis | 0.008 | -5.483 | 5.499 | 0.998 |
| Severe IVH ≧ Grade 3 | 8.031 | -3.551 | 19.613 | 0.174 |
| Necrotizing enterocolitis | 3.507 | -3.192 | 10.205 | 0.305 |
| Hemodynamically significant PDA | 0.552 | -5.215 | 6.318 | 0.851 |
| Bronchopulmonary dysplasia | 0.533 | -7.035 | 8.100 | 0.890 |
| Gestational age, weeks | 0.400 | -0.997 | 1.796 | 0.575 |
| **Eotaxin-1** |  |  |  |  |
| Late-onset sepsis | -110.923 | -276.378 | 54.532 | 0.189 |
| Severe IVH ≧ Grade 3 | 25.048 | -203.690 | 253.787 | 0.830 |
| Necrotizing enterocolitis | -218.638 | -415.802 | -21.474 | 0.030 |
| Hemodynamically significant PDA | 54.800 | -58.288 | 167.888 | 0.342 |
| Bronchopulmonary dysplasia | -53.986 | -238.163 | 130.190 | 0.566 |
| Gestational age, weeks | -14.631 | -32.328 | 3.066 | 0.105 |
| **Eotaxin-2** |  |  |  |  |
| Late-onset sepsis | -51.256 | -169.723 | 67.212 | 0.396 |
| Severe IVH ≧ Grade 3 | 109.290 | -60.769 | 279.349 | 0.208 |
| Necrotizing enterocolitis | -175.760 | -338.201 | -13.318 | 0.034 |
| Hemodynamically significant PDA | 61.918 | -42.116 | 165.953 | 0.243 |
| Bronchopulmonary dysplasia | -92.831 | -246.130 | 60.467 | 0.235 |
| Gestational age, weeks | -18.797 | -38.747 | 1.153 | 0.065 |
| **ICAM-1** |  |  |  |  |
| Late-onset sepsis | -106.133 | -416.710 | 204.443 | 0.503 |
| Severe IVH ≧ Grade 3 | 44.945 | -448.802 | 538.692 | 0.858 |
| Necrotizing enterocolitis | -510.678 | -1228.252 | 206.896 | 0.163 |
| Hemodynamically significant PDA | -23.437 | -421.767 | 374.893 | 0.908 |
| Bronchopulmonary dysplasia | -270.804 | -990.419 | 448.811 | 0.461 |
| Gestational age, weeks | -125.186 | -208.611 | -41.760 | 0.003 |
| **IL-2** |  |  |  |  |
| Late-onset sepsis | -3.497 | -14.251 | 7.257 | 0.524 |
| Severe IVH ≧ Grade 3 | 15.885 | 2.065 | 29.706 | 0.024 |
| Necrotizing enterocolitis | -16.385 | -29.314 | -3.457 | 0.013 |
| Hemodynamically significant PDA | 9.046 | -1.664 | 19.756 | 0.098 |
| Bronchopulmonary dysplasia | -1.682 | -14.192 | 10.827 | 0.792 |
| Gestational age, weeks | -0.136 | -2.422 | 2.151 | 0.908 |
| **IL-7** |  |  |  |  |
| Late-onset sepsis | -25.308 | -61.780 | 11.165 | 0.174 |
| Severe IVH ≧ Grade 3 | 81.990 | 35.776 | 128.204 | 0.001 |
| Necrotizing enterocolitis | -52.898 | -119.035 | 13.238 | 0.117 |
| Hemodynamically significant PDA | 23.702 | -0.565 | 47.970 | 0.056 |
| Bronchopulmonary dysplasia | -15.718 | -80.206 | 48.770 | 0.633 |
| Gestational age, weeks | -1.902 | -7.388 | 3.585 | 0.497 |
| **IL-11** |  |  |  |  |
| Late-onset sepsis | -29.213 | -94.034 | 35.607 | 0.377 |
| Severe IVH ≧ Grade 3 | 136.613 | 15.262 | 257.963 | 0.027 |
| Necrotizing enterocolitis | 0.740 | -85.895 | 87.375 | 0.987 |
| Hemodynamically significant PDA | 61.183 | 8.563 | 113.803 | 0.023 |
| Bronchopulmonary dysplasia | -39.687 | -115.217 | 35.844 | 0.303 |
| Gestational age, weeks | -1.062 | -10.368 | 8.245 | 0.823 |
| **IL-16** |  |  |  |  |
| Late-onset sepsis | -123.192 | -326.654 | 80.270 | 0.235 |
| Severe IVH ≧ Grade 3 | -131.178 | -371.629 | 109.273 | 0.285 |
| Necrotizing enterocolitis | -388.048 | -606.855 | -169.241 | 0.001 |
| Hemodynamically significant PDA | 109.049 | -79.680 | 297.778 | 0.257 |
| Bronchopulmonary dysplasia | -34.677 | -255.721 | 186.367 | 0.758 |
| Gestational age, weeks | -1.435 | -44.018 | 41.149 | 0.947 |
| **MIP-1α** |  |  |  |  |
| Late-onset sepsis | 7.628 | -58.901 | 74.157 | 0.822 |
| Severe IVH ≧ Grade 3 | 69.729 | -78.467 | 217.925 | 0.356 |
| Necrotizing enterocolitis | 18.013 | -44.928 | 80.953 | 0.575 |
| Hemodynamically significant PDA | 6.792 | -27.702 | 41.286 | 0.700 |
| Bronchopulmonary dysplasia | -12.251 | -74.685 | 50.184 | 0.701 |
| Gestational age, weeks | -4.605 | -12.228 | 3.018 | 0.236 |
| **MIP-1β** |  |  |  |  |
| Late-onset sepsis | 11.331 | -3.340 | 26.001 | 0.130 |
| Severe IVH ≧ Grade 3 | -3.621 | -33.862 | 26.620 | 0.814 |
| Necrotizing enterocolitis | 34.222 | 17.048 | 51.395 | <0.001 |
| Hemodynamically significant PDA | 5.204 | -5.929 | 16.338 | 0.360 |
| Bronchopulmonary dysplasia | -3.643 | -22.475 | 15.189 | 0.705 |
| Gestational age, weeks | -2.052 | -5.137 | 1.033 | 0.192 |
| **MIP-1δ** |  |  |  |  |
| Late-onset sepsis | 8.125 | -32.155 | 48.405 | 0.693 |
| Severe IVH ≧ Grade 3 | -2.932 | -76.031 | 70.166 | 0.937 |
| Necrotizing enterocolitis | -30.526 | -86.628 | 25.576 | 0.286 |
| Hemodynamically significant PDA | 18.683 | -27.623 | 64.988 | 0.429 |
| Bronchopulmonary dysplasia | 4.894 | -49.595 | 59.383 | 0.860 |
| Gestational age, weeks | -3.604 | -13.426 | 6.219 | 0.472 |
| **PDGF-BB** |  |  |  |  |
| Late-onset sepsis | -3203.141 | -7779.729 | 1373.447 | 0.170 |
| Severe IVH ≧ Grade 3 | 3797.939 | -797.042 | 8392.920 | 0.105 |
| Necrotizing enterocolitis | -9311.409 | -16798.522 | -1824.296 | 0.015 |
| Hemodynamically significant PDA | 4241.378 | 430.012 | 8052.744 | 0.029 |
| Bronchopulmonary dysplasia | -3582.949 | -11026.551 | 3860.654 | 0.345 |
| Gestational age, weeks | -604.287 | -1425.758 | 217.183 | 0.149 |
| **TIMP-2** |  |  |  |  |
| Late-onset sepsis | -91.346 | -861.852 | 679.161 | 0.816 |
| Severe IVH ≧ Grade 3 | -25.559 | -1215.766 | 1164.648 | 0.966 |
| Necrotizing enterocolitis | -704.584 | -1887.149 | 477.980 | 0.243 |
| Hemodynamically significant PDA | 324.066 | -385.250 | 1033.382 | 0.371 |
| Bronchopulmonary dysplasia | -226.764 | -1389.151 | 935.622 | 0.702 |
| Gestational age, weeks | -21.435 | -171.484 | 128.613 | 0.779 |
| **TNF-β** |  |  |  |  |
| Late-onset sepsis | -39.325 | -262.956 | 184.305 | 0.730 |
| Severe IVH ≧ Grade 3 | 149.775 | -92.971 | 392.521 | 0.227 |
| Necrotizing enterocolitis | -91.113 | -394.652 | 212.426 | 0.556 |
| Hemodynamically significant PDA | 121.441 | -123.601 | 366.484 | 0.331 |
| Bronchopulmonary dysplasia | 24.407 | -277.700 | 326.513 | 0.874 |
| Gestational age, weeks | -9.969 | -64.926 | 44.988 | 0.722 |
| **PMA 36 weeks to 38 weeks** | | | | |
| **IFN-γ** |  |  |  |  |
| Late-onset sepsis | 39.149 | -18.837 | 97.134 | 0.186 |
| Severe IVH ≧ Grade 3 | -6.093 | -86.947 | 74.762 | 0.883 |
| Necrotizing enterocolitis | -33.108 | -80.867 | 14.650 | 0.174 |
| Hemodynamically significant PDA | 12.249 | -8.420 | 32.918 | 0.245 |
| Bronchopulmonary dysplasia | -32.159 | -79.140 | 14.821 | 0.180 |
| Gestational age, weeks | 1.180 | -0.984 | 3.344 | 0.285 |
| **IL-17** |  |  |  |  |
| Late-onset sepsis | 6.135 | -6.312 | 18.581 | 0.334 |
| Severe IVH ≧ Grade 3 | 0.971 | -17.193 | 19.135 | 0.917 |
| Necrotizing enterocolitis | -2.198 | -14.711 | 10.314 | 0.731 |
| Hemodynamically significant PDA | 2.246 | -2.755 | 7.246 | 0.379 |
| Bronchopulmonary dysplasia | -4.054 | -17.031 | 8.922 | 0.540 |
| Gestational age, weeks | 0.133 | -0.993 | 1.260 | 0.816 |
| **Eotaxin-1** |  |  |  |  |
| Late-onset sepsis | -144.823 | -269.626 | -20.020 | 0.023 |
| Severe IVH ≧ Grade 3 | 108.978 | -183.164 | 401.119 | 0.465 |
| Necrotizing enterocolitis | -195.334 | -354.365 | -36.303 | 0.016 |
| Hemodynamically significant PDA | 38.202 | -36.651 | 113.055 | 0.317 |
| Bronchopulmonary dysplasia | -72.668 | -233.262 | 87.926 | 0.375 |
| Gestational age, weeks | -21.430 | -38.893 | -3.967 | 0.016 |
| **Eotaxin-2** |  |  |  |  |
| Late-onset sepsis | 18.158 | -104.769 | 141.084 | 0.772 |
| Severe IVH ≧ Grade 3 | 48.747 | -162.177 | 259.670 | 0.651 |
| Necrotizing enterocolitis | -69.257 | -191.774 | 53.260 | 0.268 |
| Hemodynamically significant PDA | 49.955 | -61.008 | 160.918 | 0.378 |
| Bronchopulmonary dysplasia | -19.776 | -138.327 | 98.775 | 0.744 |
| Gestational age, weeks | -17.296 | -39.647 | 5.054 | 0.129 |
| **ICAM-1** |  |  |  |  |
| Late-onset sepsis | -13.878 | -380.973 | 353.217 | 0.941 |
| Severe IVH ≧ Grade 3 | -209.772 | -730.244 | 310.700 | 0.430 |
| Necrotizing enterocolitis | -601.267 | -1305.655 | 103.121 | 0.094 |
| Hemodynamically significant PDA | -0.481 | -379.819 | 378.858 | 0.998 |
| Bronchopulmonary dysplasia | -294.304 | -1018.227 | 429.618 | 0.426 |
| Gestational age, weeks | -125.399 | -203.349 | -47.449 | 0.002 |
| **IL-2** |  |  |  |  |
| Late-onset sepsis | -10.655 | -20.389 | -0.921 | 0.032 |
| Severe IVH ≧ Grade 3 | 1.727 | -10.896 | 14.349 | 0.789 |
| Necrotizing enterocolitis | -19.047 | -34.824 | -3.270 | 0.018 |
| Hemodynamically significant PDA | 6.110 | -2.740 | 14.959 | 0.176 |
| Bronchopulmonary dysplasia | -0.898 | -17.375 | 15.579 | 0.915 |
| Gestational age, weeks | -1.097 | -3.888 | 1.694 | 0.441 |
| **IL-7** |  |  |  |  |
| Late-onset sepsis | -50.201 | -116.139 | 15.736 | 0.136 |
| Severe IVH ≧ Grade 3 | 108.826 | -10.707 | 228.358 | 0.074 |
| Necrotizing enterocolitis | -74.620 | -196.154 | 46.915 | 0.229 |
| Hemodynamically significant PDA | 20.728 | -8.178 | 49.633 | 0.160 |
| Bronchopulmonary dysplasia | -35.245 | -158.740 | 88.250 | 0.576 |
| Gestational age, weeks | -1.033 | -9.960 | 7.894 | 0.821 |
| **IL-11** |  |  |  |  |
| Late-onset sepsis | -106.039 | -169.097 | -42.981 | 0.001 |
| Severe IVH ≧ Grade 3 | 107.504 | -32.014 | 247.022 | 0.131 |
| Necrotizing enterocolitis | -169.471 | -251.984 | -86.957 | <0.001 |
| Hemodynamically significant PDA | 57.271 | -7.721 | 122.263 | 0.084 |
| Bronchopulmonary dysplasia | -50.924 | -124.549 | 22.700 | 0.175 |
| Gestational age, weeks | -5.773 | -19.433 | 7.886 | 0.407 |
| **IL-16** |  |  |  |  |
| Late-onset sepsis | -118.607 | -391.189 | 153.975 | 0.394 |
| Severe IVH ≧ Grade 3 | -127.881 | -406.306 | 150.545 | 0.368 |
| Necrotizing enterocolitis | -381.354 | -723.973 | -38.735 | 0.029 |
| Hemodynamically significant PDA | -14.663 | -250.345 | 221.020 | 0.903 |
| Bronchopulmonary dysplasia | -49.504 | -402.039 | 303.032 | 0.783 |
| Gestational age, weeks | 4.236 | -44.166 | 52.639 | 0.864 |
| **MIP-1α** |  |  |  |  |
| Late-onset sepsis | 56.085 | -118.338 | 230.508 | 0.529 |
| Severe IVH ≧ Grade 3 | 39.573 | -227.705 | 306.850 | 0.772 |
| Necrotizing enterocolitis | -38.855 | -127.736 | 50.026 | 0.392 |
| Hemodynamically significant PDA | 47.510 | -17.338 | 112.358 | 0.151 |
| Bronchopulmonary dysplasia | -57.594 | -145.089 | 29.902 | 0.197 |
| Gestational age, weeks | -4.885 | -11.585 | 1.814 | 0.153 |
| **MIP-1β** |  |  |  |  |
| Late-onset sepsis | 18.227 | -14.675 | 51.128 | 0.278 |
| Severe IVH ≧ Grade 3 | -14.649 | -49.537 | 20.239 | 0.411 |
| Necrotizing enterocolitis | 3.570 | -12.554 | 19.694 | 0.664 |
| Hemodynamically significant PDA | 13.582 | -2.479 | 29.643 | 0.097 |
| Bronchopulmonary dysplasia | -15.980 | -30.892 | -1.067 | 0.036 |
| Gestational age, weeks | -3.400 | -5.860 | -0.941 | 0.007 |
| **MIP-1δ** |  |  |  |  |
| Late-onset sepsis | 15.470 | -13.781 | 44.721 | 0.300 |
| Severe IVH ≧ Grade 3 | -47.947 | -104.324 | 8.430 | 0.096 |
| Necrotizing enterocolitis | -26.901 | -84.580 | 30.777 | 0.361 |
| Hemodynamically significant PDA | 29.492 | -15.759 | 74.744 | 0.201 |
| Bronchopulmonary dysplasia | -10.427 | -81.730 | 60.876 | 0.774 |
| Gestational age, weeks | -7.227 | -19.817 | 5.362 | 0.261 |
| **PDGF-BB** |  |  |  |  |
| Late-onset sepsis | -2733.669 | -6638.066 | 1170.728 | 0.170 |
| Severe IVH ≧ Grade 3 | 2118.392 | -2974.472 | 7211.257 | 0.415 |
| Necrotizing enterocolitis | -7686.226 | -14073.601 | -1298.851 | 0.018 |
| Hemodynamically significant PDA | 2679.173 | -764.066 | 6122.413 | 0.127 |
| Bronchopulmonary dysplasia | -2492.584 | -9875.397 | 4890.229 | 0.508 |
| Gestational age, weeks | -502.248 | -1494.507 | 490.010 | 0.321 |
| **TIMP-2** |  |  |  |  |
| Late-onset sepsis | -302.857 | -882.934 | 277.220 | 0.306 |
| Severe IVH ≧ Grade 3 | -520.460 | -1416.717 | 375.798 | 0.255 |
| Necrotizing enterocolitis | -1202.051 | -2168.800 | -235.302 | 0.015 |
| Hemodynamically significant PDA | 349.676 | -229.542 | 928.893 | 0.237 |
| Bronchopulmonary dysplasia | 32.496 | -963.342 | 1028.334 | 0.949 |
| Gestational age, weeks | -53.981 | -185.346 | 77.385 | 0.421 |
| **TNF-β** |  |  |  |  |
| Late-onset sepsis | -147.737 | -419.030 | 123.557 | 0.286 |
| Severe IVH ≧ Grade 3 | 57.233 | -244.129 | 358.595 | 0.710 |
| Necrotizing enterocolitis | -318.685 | -783.861 | 146.491 | 0.179 |
| Hemodynamically significant PDA | 50.514 | -193.040 | 294.068 | 0.684 |
| Bronchopulmonary dysplasia | -12.337 | -493.716 | 469.041 | 0.960 |
| Gestational age, weeks | -22.070 | -83.238 | 39.098 | 0.479 |
